# CTX-M-type ESBL-mediated resistance to third-generation cephalosporins and conjugative transfer of resistance in Gram-negative bacteria isolated from hospitals in Tamil Nadu, India

**DOI:** 10.1099/acmi.0.000142

**Published:** 2020-06-11

**Authors:** Ramesh Nachimuthu, Velu Rajesh Kannan, Bulent Bozdogan, Vaithilingam Krishnakumar, Karutha Pandian S, Prasanth Manohar

**Affiliations:** ^1^​ Antibiotic Resistance and Phage Therapy Laboratory, School of Bio Sciences and Technology, Vellore Institute of Technology (VIT), Vellore-632014, India; ^2^​ Department of Microbiology, Bharathidasan University, Tiruchirappalli, Tamil Nadu, India; ^3^​ Medical Faculty, Medical Microbiology Department, Adnan Menderes University, Aydin, Turkey; ^4^​ Department of Biotechnology, Alagappa university, Karaikudi, Tamil Nadu, India; ^5^​ Zhejiang University-University of Edinburgh (ZJU-UoE) Institute, Zhejiang University, International Campus, PR China, Haining, Zhejiang; ^6^​ School of Medicine, The Second Affiliated Hospital Zhejiang University (SAHZU),, Hangzhou, Zhejiang, PR China

**Keywords:** CTX-M, cephalosporins, cefotaxime, ESBL, plasmid-borne resistance, transconjugation

## Abstract

Clinical pathogens, especially Gram-negative bacteria developing resistance to third-generation cephalosporins, are making clinical outcomes more complicated and serious. This study was undertaken to evaluate the distribution of CTX-M-type extended-spectrum β-lactamases (ESBLs) in Tamil Nadu, India. For this study, clinical samples were collected from five different hospitals located in Tamil Nadu and the ESBL-producing Gram-negative isolates were characterized. MIC was performed using cefotaxime and ceftazidime. The *bla*_ESBL_-producing genes were screened using multiplex PCR for the genes, CTX-M group-1, -2, -8, -9, -26. The conjugation studies were performed using *Escherichia coli* AB1157 as a recipient for the isolates harbouring plasmid-borne resistance following broth-mating experiment. In total, 1500 samples were collected and 599 Gram-negative bacteria were isolated that included *E. coli* (*n*=233), *Klebsiella pneumoniae* (*n*=182), *Pseudomonas aeruginosa* (*n*=79), *Citrobacter* spp. (*n*=30), *Proteus mirabilis* (*n*=28), *Salmonella* spp. (*n*=21), *Acinetobacter baumannii* (*n*=12), *Serratia spp*. (*n*=6), *Shigella* spp. (*n*=4), *Morganella morganii* (*n*=3) and *Providencia* spp. (*n*=1). MIC results showed that 358 isolates were resistant to cefotaxime and ceftazidime. Further, ESBL gene-amplification results showed that 19 isolates had CTX-M group-1 gene including *E. coli* (*n*=16)*, K. pneumoniae* (*n*=2) and *P. aeruginosa* (*n*=1) whereas one *M. morganii* isolate had CTX-M group-9, which was plasmid-borne. Through conjugation studies, 12/20 isolates were found to be involved in the transformation of its plasmid-borne resistance gene. Our study highlighted the importance of horizontal gene transfer in the dissemination of plasmid-borne *bla*_CTX-M-type_ resistance genes among the clinical isolates.

## Introduction

The arousal of β-lactamase production in bacteria provoked the emergence of β-lactam resistance. β-lactamase is an enzyme, which actively participates in the hydrolysis of β-lactam antibiotics; as a consequence, it loses its antibacterial activity against the pathogen [1]. ESBL-producing Gram-negative bacteria are rapidly evolving and are the up-most clinical vexation by considering the potential risk of disseminating the infection [2]. The infections caused by such resistant bacteria have been increasing over the last decade and has become a worldwide epidemic. Cephalosporin-resistant bacteria were also found to have co-resistance to other antibiotics such as tetracyclines, sulfonamides and aminoglycosides, posing serious therapeutic challenge [3]. Among the β-lactam antibiotics, the third-generation cephalosporin plays a vital role in treating serious infections caused by Gram-negative bacteria [4]. The antibacterial activity of the cephalosporin group of antibiotics was inhibited greatly by the production of ESBLs. In the *Enterobacteriaceae* family, *E. coli* and *K. pneumoniae* were identified as major ESBL-producing organisms. This enzyme is also effectively produced by other members of the *Enterobacteriaceae* and certainly by non-fermenting Gram-negative bacteria [5]. The concern towards ESBL-producing *Klebsiella* has increased after the report from Germany that revealed there is an emergence of plasmid-mediated resistance to ESBL and was later reported in India and France [6, 7]. Urinary tract infections caused by ESBL-producing *E. coli* and *K. pneumoniae* are of great concern because of the lesser availability of last-line treatment options [8]. CTX-M emerge as the prevalent member of the ESBL family, which are actively involved in imparting resistance to the third/fourth generation of the cephalosporin group of antibiotics [9]. Among the various CTX-M variants, CTX-M-15 was considered to be the more prominent and it was first reported from the Indian subcontinent [10]. The emanation of plasmid- mediated resistance, integrons and other mobile genetic elements made chances for the spread of resistance genes rapidly through horizontal gene transfer. The mobilization of resistance genes was greatly encouraged by the various genetic elements like insertion sequences, a segment that provides a switch around mechanism of resistance to the bacteria for their survival. The evolution of bacteria was highly supported by horizontal gene transfer (HGT) by taking up the genes or operons from the pool of mobile genetic elements [11]. More studies on the prevalence of ESBL producers among nosocomial pathogens and plasmid-mediated resistance transmissibility will provide insight into the dissemination of resistance in the clinical settings. Our study aims to find out the prevalence of CTX-M-type ESBL producers among Gram-negative bacteria isolated from clinical samples in Tamil Nadu, South India and also to evaluate the role of bacterial conjugation in disseminating plasmid-borne *bla*_CTX-M_ gene.

## Methods

### Clinical samples and study area

**Fig. 1.**
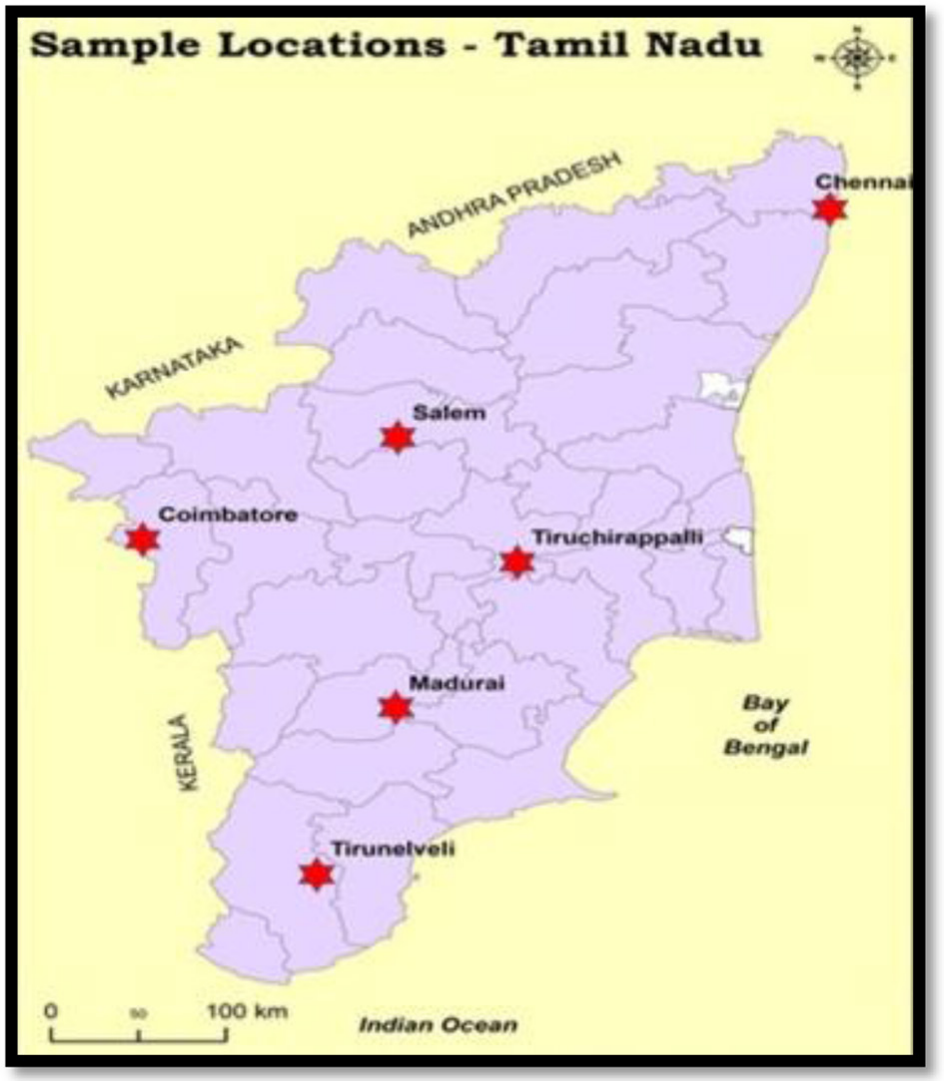
Sampling locations: five different regions were selected in Tamil Nadu for sample collection.

A total of 1500 samples (urine, blood, pus, stool, ET secretion, sputum, semen, vaginal swab, cerebrospinal fluid, throat, swab, wound swab, tracheoscopy, bronchial wash, catheter tip and pleural fluid) were collected from patients during the period of 2008 to 2010. The clinical samples were obtained from various locations in Tamil Nadu from both male and female patients between the age group of 3 to 60 years. The inclusion criteria were both community-associated and hospital-acquired infections. The inpatient and outpatient samples from the microbiology laboratories of Billroth Hospital (Chennai), Viva Laboratory (Salem), Miritasanjivini Laboratory (Coimbatore), Bose Laboratory (Madurai), KAPV Medical College (Tiruchirappalli) and Annai Vellankanni Hospital (Tirunelveli) were collected (Fig. 1). This study was approved by the Institutional Ethics Committee for Human Research (IEC) No: DM/2010/101/23 project number-4.

### Isolation and identification of clinical isolates

The samples were collected in sterile containers and were cultured immediately on blood agar and characterized on MacConkey agar plates. Using standard microbiological procedures, the clinically important bacteria were isolated from the respective samples [12]. Bacterial isolation mainly focused on *Enterobacteriaceae* and all the isolated strains were identified at species level using automated methods, API 20E identification system (BioMerieux, Marcy, Etoile, France) and further identified using the standardized microbial identification by ID 32GN strips.

### Antibiotic susceptibility testing

#### Disc-diffusion method

Antibiotic susceptibility testing was performed for all the isolates by disc-diffusion method and the results were elucidated according to Clinical and Laboratory Standards Institute (CLSI) guidelines, 2009 [13]. The following antibiotics were used, ampicillin (10 µg), amikacin (30 µg), gentamicin (10 µg), ciprofloxacin (30 µg), ofloxacin (5 µg), cefotaxime (30 µg), ceftazidime (30 µg), ceftriaxone (30 µg), cefpodoxime (10 µg), cefepime (30 µg), aztreonam (30 µg), imipenem (10 µg), meropenem (10 µg), piperacillin-tazobactam (100/10 µg), amoxicillin-clavulanic acid (20/10 µg) and nitrofurantoin (30 µg) (Hi-Media, India). The reference strains used for this study were *E. coli* ATCC25988, *K. pneumoniae* ATCC700603 and *P. aeruginosa* ATCC27 853. ESBL enzyme-producing isolates were determined by the combination-disc technique using the test inoculum of 10^5^ c.f.u. ml^−1^. The antibiotic discs containing ceftazidime (30 µg), cefotaxime (30 µg) and cefpodoxime (10 µg) were tested alone or in combination with clavulanic acid (10 µg). Those isolates showed an increase in their inhibitory zones, when tested against ceftazidime-clavulanate, cefotaxime-clavulanate or cefpodoxime-clavulanate of at least >5 mm when compared to ceftazidime, cefotaxime or cefpodoxime alone respectively, were determined to be ESBL producers [14].

#### MIC

MIC was performed with micro-broth dilution method for ceftazidime and cefotaxime following the CLSI guidelines, 2009 [13]. In brief, twofold serial dilutions were done by adding 100 µl of MH broth in a 96-well plate and the respective antibiotics were amended with the concentration ranging from 0.25 to 256 mg l^−1^, respectively. Later, an inoculum of 10^5^ c.f.u. ml^−1^ was inoculated on the respective wells and incubated for 16 h. For this study, *K. pneumoniae* ATCC700603 and *E. coli* ATCC25988 were used as positive and negative controls, respectively.

### Molecular studies

#### DNA isolation

DNA was obtained using a modified alkaline lysis method. Briefly, a single colony of each bacterium was inoculated from MacConkey agar into 5 ml of LB broth and incubated at 37 °C for 18 h. Cells were pelleted and resuspended in 100 µl of glucose 50 mM, 25 mM tris-Cl and 10 mM EDTA (pH 8.0), and RNase was added to a final concentration of 20 µg ml^−1^. To the solution, 200 µl of 0.2 N NaOH, 1 % SDS was added. In the tubes, 150 µL of 5M potassium acetate, glacial acetic acid and water was added and stored at 4 °C for 5 min and centrifuged. The supernatant was extracted with two volumes of phenol: chloroform. The nucleic acid was precipitated by adding 100 % ethanol. The pellet was washed with 70 % ethanol and nucleic acid was recovered by centrifugation; the DNA pellet was resuspended in sterile distilled water and stored at 4 °C. The plasmid profile of each strain was observed in 0.8 % of agarose gel.

#### PCR

The amplification of genes coding for *bla*_CTX-M_ was performed by PCR using the primers reported in our previous study [15]. PCR was accomplished in 50 µl reactions, approximately 10 ng of plasmid DNA as a template, reaction volume containing: 10 pmol of each primer, 200 µm dNTP, 1.5 mM MgCl_2_, 1X Taq buffer and 2U of Taq polymerase (Genei, Bangalore, India). All the PCR products were sequenced.

### Transferability assay

To study the transferability of resistance, mating experiments were performed using plasmid-free *E. coli* AB1157 (Str^r^) as the recipient strain and all the ESBL-producing *E. coli* as donors [16]. Overnight cultures of both donor and recipient strains grown in MH broth at 37 °C were mixed together at 1 : 10 (v/v) proportion and incubated at 37 °C for at least 4 h without shaking. Then, 0.1 ml of the mixture was spread onto the surface of MH agar plates containing streptomycin (100 mg l^−1^) and cefotaxime (2 mg l^−1^). The transconjugants growing on the selection plates were subjected to an ESBL screening, antibiotic susceptibility testing and PCR to confirm the possible acquisition of resistance.

### Statistical analysis

Data were recorded and entered into a Graph Pad software (online) database. Analyses were performed using Chi-square test, or Fisher's exact test when appropriate to compare proportions. All statistical analyses were two-sided, and significance was set at *P*<0.05.

## Results

### Isolation of Gram-negative bacteria from clinical samples

**Fig. 2.**
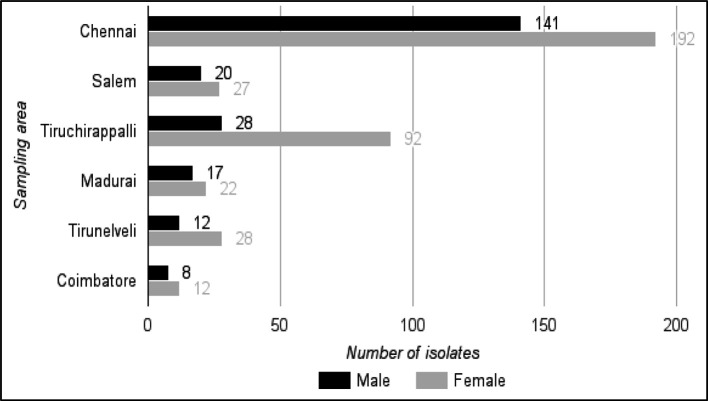
Distribution of Gram-negative bacteria among male and female patients in the five sampling locations.

For this prevalence study, a total of 1500 samples were collected and 599 (39.9 %) Gram-negative bacteria were isolated (Table 1). The clinical distribution of isolates includes, 346 (57.7 %) from urine, 80 (13.3 %) from stool, 55 (9.1 %) from pus, 44 (7.3 %) from blood, 31 (5.1 %) from wound swab, 17 (2.8 %) from sputum, 10 (1.6 %) from endotracheal secretion, 4 (0.6 %) from throat swab, 3 (0.5 %) from bronchial wash, 2 (0.3 %) from semen, 2 (0.3 %) from vaginal swab, 2 (0.3 %) from cerebrospinal fluid, 2 (0.3 %) from tracheoscopy, 1 (0.1 %) each from catheter tip and pleural fluids. From the samples, a total of 226 isolates from male and 373 from female patients were isolated (Fig. 2).

**Table 1.** The prevalence rate of Gram-negative bacteria isolated from the clinical samples between different age groups and sex

Clinical sample	Age group	Distribution of clinical samples and isolates						Total no. of samples	Total no. of isolates
		Samples			Isolates				
		Male	Female	*P-* value	Male	Female	*P-* value		
Urine	Below 15	80	90	0.087	38	49	0.0001	815	346
	b/w15-45	172	163		91	96			
	Above 45	138	172		32	40			
Blood	Below 15	17	11	0.193	2	7	0.076	126	44
	b/w15-45	41	22		6	18			
	Above 45	12	23		3	8			
Pus	Below 15	8	13	0.441	6	9	0.190	152	55
	b/w15-45	44	36		8	15			
	Above 45	19	32		2	15			
Stool	Below 15	21	21	0.399	5	18	0.025	195	80
	b/w15-45	53	42		10	31			
	Above 45	29	29		6	10			
Endotracheal secretion	Below 15	3	2	0.843	1	2	0.333	25	10
	b/w15-45	6	7		1	6			
	Above 45	4	3		0	0			
Sputum	Below 15	9	6	0.885	2	3	0.433	49	16
	b/w15-45	11	13		1	5			
	Above 45	5	5		1	4			
Semen	Below 15	1	2	0.185	0	2	0.529	20	2
	b/w15-45	7	4		0	0			
	Above 45	5	1		0	0			
Vaginal swab	Below 15	0	1	0.725	0	0	1.000	8	2
	b/w15-45	1	3		1	0			
	Above 45	2	1		0	1			
Cerebrospinal fluid	Below 15	1	0	0.499	0	0	0.529	1	2
	b/w15-45	0	0		0	1			
	Above 45	0	0		0	1			
Throat swab	Below 15	2	2	0.813	0	1	1.000	17	4
	b/w15-45	3	4		0	1			
	Above 45	4	2		1	1			
Wound swab	Below 15	11	9	0.409	2	7	0.185	75	31
	b/w15-45	16	17		2	12			
	Above 45	14	8		4	4			
Tracheoscopy	Below 15	1	0	0.373	0	1	0.529	4	2
	b/w15-45	2	1		0	1			
	Above 45	0	0		0	0			
Bronchial wash	Below 15	3	2	0.506	1	2	1.000	8	3
	b/w15-45	1	1		0	0			
	Above 45	1	0		0	0			
Catheter tip	Below 15	0	1	1.000	0	1	0.529	5	2
	b/w15-45	2	0		0	0			
	Above 45	0	2		0	0			

*P*-value = male vs female. b/w, between. *P*<0.05 was considered as statistically significant.

### Identification of clinical isolates

The isolates belonged to 11 different genera of Gram-negative bacteria including *E. coli* (*n*=233, 38 %), *K. pneumoniae* (*n*=182, 30 %), *P. aeruginosa* (*n*=79, 13 %), *Citrobacter* spp. (*n*=30, 5 %), *Proteus mirabilis* (*n*=28, 4 %), *Salmonella* spp. (*n*=21, 3 %), *A. baumannii* (*n*=12, 2 %), *Serratia* spp. (*n*=6, 1 %), *Shigella* spp. (*n*=4, 0.6 %), *Morganella morganii* (*n*=3, 0.5 %) and *Providencia* spp. (*n*=1, 0.1 %). Among the isolates, *E. coli* and *K. pneumoniae* were the most predominant species in all the six sampling locations and the majority of the strains were obtained from urine samples followed by stool and pus. The maximum number of the aforementioned Gram-negative bacteria was isolated from the patients between the age group of 15 to 45; in addition, samples collected from females were found to encounter pathogenic Gram-negative bacteria more frequently than from the male patients.

### Antibiotic susceptibility testing

Among the bacterial pathogens isolated from the clinical samples from various locations in Tamil Nadu, 498 (83.1 %) out of 599 strains were found to be cephalosporin-resistant by disc-diffusion studies. The antimicrobial sensitivity patterns showed that the overall resistance pattern for ampicillin was 87.5 % followed by cefepime (82.5 %), cefotaxime (79.2 %), ceftriaxone (78.2 %), ceftazidime (77.5 %), cefpodoxime (76.5 %), ofloxacin (70.8 %), gentamicin (67.7 %), ciprofloxacin (64.5 %), amikacin (57.3 %), nitrofurantoin (26.5 %), piperacillin-tazobactam (21.8 %), amoxicillin-clavulanic acid (21.5 %), imipenem (3.9 %) and meropenem (3.6 %). The overall sensitivity for meropenem was 96.4 %, followed by imipenem (96.1 %), amoxicillin-clavulanic acid (78.5 %) and piperacillin-tazobactam (78.1 %). The overall prevalence of isolates resistant to cephalosporins was 84.3, 72.3, 81.6, 79.4, 95 and 80 % in Chennai, Salem, Tiruchirappalli, Madurai, Tirunelveli and Coimbatore, respectively. In the case of combination-disc method, out of 498 isolates screened, only 358 (64.7 %) isolates were positive and 140 (35.2 %) isolates were negative for ESBL production (Table 2).

**Table 2.** Comparison of two different phenotypic test results used to identify the ESBL producer

Sampling location	Distribution of ESBL producer-phenotypic analysis			
	By disc-diffusion test		By combination-disc method*	
	Total isolates	ESBL producer	Total isolates	ESBL producer
**Chennai**	333	281 (84.3)	281	188 (66.9)
**Salem**	47	34 (72.3)	34	34 (100)
**Tiruchirappalli**	120	98 (81.6)	98	79 (88.7)
**Madurai**	39	31 (79.4)	31	28 (90.3)
**Tirunelveli**	40	38 (95)	38	22 (57.8)
**Coimbatore**	20	16 (80)	16	07 (43.7)
**Total (%**)	599	498 (83.1)	498	358 (71.8)

*Bacterial isolates that were found to be a ESBL producer using the disc-diffusion method was further studied for the combination-disc method.

MIC was performed for those isolates that were found to be cephalosporin-resistant by disc-diffusion and combination-disc methods. Of the 358 isolates screened for the MIC analysis using ceftazidime and cefotaxime, 85 % of the isolates showed MIC ≥64 µg ml^−1^. However, cefotaxime- and ceftazidime-resistant isolates with MIC values >256 µg ml^−1^ were 36 and 40, respectively (Table 3). In total, 305 isolates were found to be cefotaxime- and ceftazidime-resistant through MIC results that included *E. coli* (*n*=127), *K.pneumoniae* (*n*=93), *Pseudomonas aeruginosa* (*n*=36), *Citrobacter* spp. (*n*=14), *Proteus mirabilis* (*n*=15), *Salmonella* spp. (*n*=9), *Acinetobacter baumannii* (*n*=5), *Serratia* spp. (*n*=3), *Shigella* spp. (*n*=2) and *Morganella morganii* (*n*=1).

**Table 3.** Minimal Inhibitory Concentration (MIC) results of the isolates as determined using cefotaxime and ceftazidime

Sampling location	MIC results as obtained by micro-broth dilution method*			
	Cefotaxime (≤ 256 µg ml^−1^)	Cefotaxime (≥ 256 µg ml^−1^)	Ceftazidime (≤ 256 µg ml^−1^)	Ceftazidime (≥ 256 µg ml^−1^)
**Chennai (*n*=188**)	170	18	175	9
**Salem (*n*=34)**	28	4	25	7
**Tiruchirappalli (*n*=79)**	70	8	68	13
**Madurai (*n*=28)**	28	2	27	1
**Tirunelveli (*n*=22)**	21	2	19	6
**Coimbatore (*n*=7)**	5	2	4	4
**Total (%)**	322 (89.9)	36 (10.4)	318 (88.8)	40 (11.4)
	358		358	

*Bacterial isolates that were found to be ESBL producers by earlier phenotypic studies were taken for MIC determination.

### Plasmid profiling and multiplex PCR

From the antibiotic susceptibility studies, 40 isolates that had MIC values >256 µg ml^−1^ were selected for plasmid profiling. Out of 40 selected isolates, 28 (70 %) yielded plasmids of the size ranging from 1 to 100 kb while the remaining were found to be plasmid-free. Multiple plasmids were seen in *E. coli, K. pneumoniae* and *P. aeruginosa *. There were some isolates that harboured six or seven plasmids of varying size, for instance, *E. coli* isolated from a urine sample carried seven plasmids and other *E. coli* had six plasmids, and *K. pneumoniae* with five plasmids were isolated from stool samples. Therefore, *E. coli* and *K. pneumoniae* were the most predominant with more than two plasmids of varying size. All the isolates that had one or more plasmids were subjected to conjugation experiments.

Plasmid DNA was used to screen CTX-M group genes (1, 2, 8, 9 and 26) and our data revealed that group-1 CTX-M was predominantly found in all the sampling locations included in the study. CTX-M-group-1, which was the most prevalent ESBL gene, was detected in 19 isolates (95 % of ESBLs), including 16 (80 %) of *E. coli *, 2 (10 %) of *K. pneumoniae *, 1 (5 %) of *P. aeruginosa* and followed by group-9 in one isolate (5 %), in *M. morganii *. More than one CTX-M β-lactamases (group-1, -9) were found in one *E. coli* strain. None of the isolates from any of the locations had shown the presence of all the five CTX-M group genes. All the amplified CTX-M group (1 and 9) genes were sequenced and GenBank accession numbers were obtained.

### Transferability assay

From the molecular studies, 20 isolates that were found to be carrying plasmid-borne CTX-M genes were selected for broth-mating experiments. Among the 20 isolates, 12 were found to be involved in conjugating plasmid-borne *bla*_CTX-M_ resistance to *E. coli* AB1157 at the frequency of 10^5^. Further, the conjugated plasmids were amplified by multiplex PCR for the presence of CTX-M gene in each transconjugant. As expected, among the 12 positive isolates, 11 isolates were found to carry CTX-M group 1 gene and one isolate was found to carry group 9. The 12 isolates that were involved in transferability includes, eight *E. coli *, two *K. pneumoniae *, one each of *P. aeruginosa* and *M. morganii *.

## Discussion

The prevalence of CTX-M-type ESBL producers has progressively increased throughout healthcare settings, though it may vary between geographical locations [17–19]. The highest incidence of cephalosporin resistance is recorded particularly among *E. coli *, *K. pneumoniae *, *P. aeruginosa *, which are known to cause a wide range of hospital-acquired infections [20, 21]. The prevalence of ESBL-mediated infections are very high in India, especially nosocomial infections [22]. The rate of ESBL-mediated resistance in the hospitals of India is found to be 60–77 % [21]. Infections due to ESBL-producing strains are infamous for unfavourable clinical outcomes, treatment failure and high mortality rates. Our study showed that *K. pneumoniae* was the most common (56 %, *n*=75) ESBL producer from the sampling locations, followed by *P. aeruginosa* with 29 % (*n*=39), and *E. coli* with 15 % (*n*=20). Basavaraj *et al.*, have reported that, out of 218 *Enterobacteriaceae* isolates, *E. coli* (57.8 %) was most prevalent, followed by *K. pneumoniae* (25.6%), *Citrobacter* sp. (6.5 %), *Proteus* sp. (6.5 %), *Salmonella* sp. (1.8 %) and *Enterobacter* sp. (1.8 %) [23]. In this study, the patients between the age groups of 15 to 45 were most predominantly infected with Gram-negative ESBL-producing isolates. The study also recorded that the female patients were most frequently infected than the male patients in all six sampling locations of Tamil Nadu. Another study, led by Muraleetharan *et al.*, has shown the increased incidence of ESBL-producing Uropathogenic *E. coli* (UPEC) among female patients (56 %) above 35 years in Tamil nadu, India [24]. Similarly, in another study, female patients were found to be infected with ESBL-producing *E. coli* [25]. The majority of the patients isolated with ESBL-producing isolates were over 60 years old and this may be due to the increased hospitalization of the patients in ICUs. This shows that the antibiotic resistance in Gram-negative bacteria has built up progressively during the last few decades, leading to the increased incidence of outbreaks in infections due to the existence of multi-drug-resistant (MDR) bacteria [26].

Our study showed the high prevalence of ampicillin-resistant Gram-negative isolates, mainly, due to the overuse of ampicillin in the study region. Followed by cephalosporins such as cefepime, cefotaxime, ceftriaxone, ceftazidime, cefpodoxime, ofloxacin, gentamicin, ciprofloxacin. Though third-generaion cephalosporins are commonly used during the study period, the resistant strains are due to over the counter drugs. The overall sensitivity to meropenem was 96.4 % followed by imipenem 96.1 %, amoxicillin-clavulanic acid (78.5 %) and piperacillin-tazobactam (78.1 %) and this correlates very well with the findings of the earlier studies in the same geographical region [24]. As carbapenems are one of the last resort antibiotics, they have restricted use. The infection due to the ESBL enzymes can be overcome by the use of β-lactamase inhibitors such as clavulanic acid and sulbactum, which can be an effective alternative to the ESBL-mediated resistance [27]. One prospective study had shown that *Enterobacteriaceae* isolates were resistant to cefotaxime (100%), ceftazidime (76 %), cefepime (71 %) and aztreonam (42 %). A study by Senthamarai *et al.*, showed the less resistance to ceftazidime (65.38 %) and cefotaxime (51.92 %) when compared to our study [27]. Shahzad *et al.* found that the most prevalent isolates *E. coli* (58.6 %), *Klebsiella* sp. (32.9 %) and *Pseudomonas* (8.6 %) were resistant to most of the antibiotics including cefazolin, ceftriaxone, cefuroxime, ampicillin and co-trimoxazole but sensitive to imipenem and meropenem [28]. Our study found that out of 358 strains screened for the MICs of ceftazidime and cefotaxime, 36 (10 %) isolates had MIC >256 µg ml^−1^. A study by Bente-Olesen *et al*. showed that, out of 115 ESBL isolates, 92 % produced CTX-M enzymes, most commonly CTX-M-15 (52 %), but also CTX-M-14 (19 %), CTX-M-1 (11 %) and CTX-M-27 (5 %) [29]. Our present investigation also confirmed that the isolates from Chennai were positive for group-1 and group-9 CTX-M. The CTX-M-group-1 was the most predominant ESBL detected in 19 isolates (95 % of ESBL) followed by group-9 and this was in accordance with another study, which detected *bla*_CTX-M-1_ and *bla*_CTX-M-9_ at 66.9 % (87/130) and 54.6 %(71/130) respectively, whereas none of the isolates in this study was positive for *bla*_CTX-M-2_, *bla*_CTX-M-8_ and *bla*_CTX-M-25_ groups [30].

Further, the transfer of ESBL genes via horizontal gene transfer is a worrisome problem that mainly leads to the dissemination of resistance. The *bla*_CTX-M_ is known to be the highest disseminated ESBL-resistant gene, while *bla*_GES_ and *bla*_TEM_ are found to have the lowest dissemination rate [31]. Our study proved that Gram-negative bacteria have been involved in the conjugative transfer of plasmid-borne CTX-M genes. As studied, the isolates of *E. coli, K. pneumoniae, E. cloacae* and *M. morganii* were found to be involved in conjugating its resistance to recipient *E. coli* AB1157. Silva-Sanchez *et al.* had assayed 104 isolates for mating, of which 68.4 % of the isolates were successful, corresponding to 64 % of Kp-ESBL 68 % of Ecl-ESBL and 81 % of Ecl-ESBL [32]. In general, most transconjugants had acquired cefotaxime resistance through the larger plasmids (70 kb) carrying ESBL-encoded genes that were found in the respective clinical isolates. In addition, these transconjugants co-expressed other antibiotic resistance genes such as aminoglycosides (kanamycin and gentamicin), chloramphenicol and tetracycline. From our study, it is clear that there is a sharp rise in the resistance among *Enterobacteriaceae,* especially ESBL-mediated. One of the main reasons for the dissemination of ESBL-mediated resistance is horizontal gene transfer. The increasing antibiotic resistance in developing countries is due to the misuse and oversue of antibiotics in humans and animal husbandry. Other factors being poor hygiene and the lack of awareness. Thus, there is an urgent need to prevent the uncontrolled use of antibiotics and to control the growing antibiotic resistance.

## Conclusion

This study concludes that there is a high prevalence of ESBL producers in the regions of Tamil Nadu, India. In addition, it highlights the existence of *bla*_CTX- M_ group-1-type ESBL producers among the Gram-negative bacteria. The ability of these bacterial strains to transfer plasmid-borne resistance genes to the different bacteria has become a problem of great concern in hospital settings. Thus, this study sheds light on the need for routine clinical detection of ESBL producers and the importance of preventive measures in hospitals to prevent the spread of antibiotic-resistant bacterial infections.
